# Pure-Shift-Based Proton Magnetic Resonance Spectroscopy for High-Resolution Studies of Biological Samples

**DOI:** 10.3390/ijms25094698

**Published:** 2024-04-25

**Authors:** Haolin Zhan, Yulei Chen, Yinping Cui, Yunsong Zeng, Xiaozhen Feng, Chunhua Tan, Chengda Huang, Enping Lin, Yuqing Huang, Zhong Chen

**Affiliations:** 1 Department of Electronic Science, Fujian Provincial Key Laboratory of Plasma and Magnetic Resonance, State Key Laboratory of Physical Chemistry of Solid Surfaces, Xiamen University, Xiamen 361005, China; 2 Department of Biomedical Engineering, Anhui Provincial Engineering Research Center of Semiconductor Inspection Technology and Instrument, Anhui Province Key Laboratory of Measuring Theory and Precision Instrument, Hefei University of Technology, Hefei 230009, China

**Keywords:** magnetic resonance spectroscopy, ISIS localization, pure shift, spectral congestion, biological samples

## Abstract

Proton magnetic resonance spectroscopy (^1^H MRS) presents a powerful tool for revealing molecular-level metabolite information, complementary to the anatomical insight delivered by magnetic resonance imaging (MRI), thus playing a significant role in in vivo/in vitro biological studies. However, its further applications are generally confined by spectral congestion caused by numerous biological metabolites contained within the limited proton frequency range. Herein, we propose a pure-shift-based ^1^H localized MRS method as a proof of concept for high-resolution studies of biological samples. Benefitting from the spectral simplification from multiplets to singlet peaks, this method addresses the challenge of spectral congestion encountered in conventional MRS experiments and facilitates metabolite analysis from crowded NMR resonances. The performance of the proposed pure-shift ^1^H MRS method is demonstrated on different kinds of samples, including brain metabolite phantom and in vitro biological samples of intact pig brain tissue and grape tissue, using a 7.0 T animal MRI scanner. This proposed MRS method is readily implemented in common commercial NMR/MRI instruments because of its generally adopted pulse-sequence modules. Therefore, this study takes a meaningful step for MRS studies toward potential applications in metabolite analysis and disease diagnosis.

## 1. Introduction

Magnetic resonance spectroscopy (MRS) provides a powerful tool to understand biological samples by noninvasively recording molecular-level information regarding biochemical compositions and related molecular structures. Its efficacy is widely demonstrated in biological applications, disease detections, and clinical diagnostics [1,2,3,4], where the molecular-level metabolite information is provided as a complement to the anatomical insight delivered by magnetic resonance imaging (MRI) [5]. In detail, MRS spectra are acquired from localized cube volumes of interest referring to MRI images, thus offering the unique connection between metabolite information and related anatomical structure. According to the subsequent analysis from acquired MRS spectra, it allows one to perform biochemical evaluation related to physiologic function, disease impact, and clinical treatment, thus boosting MRS application landscapes in metabolite studies and disease diagnosis [6,7].

For MRS experiments, it is a prerequisite to accurately locate targeted volumes of interest and exclude all space regions outside the selected volumes. Accordingly, a group of MRS methods have been established based on different spatially localized techniques [7,8], which commonly combine the use of selective radio frequency (RF) pulses and three mutually orthogonal slice-selective gradients. Two typical MRS protocols, STEAM [9] and PRESS [10,11], are developed for localized MRS measurements based on the stimulated echo and spin echo evolution, respectively. Nevertheless, the effects of *T*_2_-relaxation decay and *J*-coupling modulation during the spatial localization process inevitably cause signal attenuations and peak distortions, and it may hinder accurate metabolite measurements in case of long echo times. Another MRS method, image-selected in vivo spectroscopy (ISIS) [12,13,14], selects targeted volumes prior to signal excitation from eight transition experiments. This implementation makes acquired MRS signals mainly dependent on *T*_1_ relaxation rather than *T*_2_ relaxation, thus more suitable for measurements of biological tissue samples with short *T*_2_-relaxation properties. Additionally, the LASER methods [15,16], which adopt three pairs of adiabatic full-passage pulses to replace the original selective pulse for accurate signal selection of three orthogonal slices in space, are proposed to overcome possible irregular volume shapes of selected spins caused by RF-field (B1) inhomogeneity and yield satisfactory MRS results under nonideal RF-field conditions.

Because of high natural abundance and superior detection sensitivity, proton (^1^H) is commonly used in practical MRS measurements, along with methodologic developments and groundbreaking applications in basic and clinical studies. However, ^1^H MRS measurements of biological samples generally encounter the challenge of spectral congestion with peak crowding and even overlapping caused by numerous biological metabolites dispersed in the limited proton frequency range. Despite benefitting metabolite identification, *J* couplings still aggravate the dilemma of spectral congestion. In general, most metabolites contain coupled proton spin systems, and they would yield extensive *J*-coupling splittings along with multiplet peaks observed in the acquired MRS spectra, which further leads to peak crowding and overlapping and significantly hinders metabolite identification and quantification.

Advanced MRI scanners with higher magnetic fields serve as a direct way to deal with the challenge of spectral congestion by expanding chemical shift dispersion and improving signal intensity. However, the achievements of high magnetic fields are accompanied by expensive economic costs and tremendous technical challenges; thus, the benefits from high magnetic fields are generally limited in practical NMR-related experiments. Two-dimensional (2D) MRS spectroscopy [17,18,19], as well as its ultrafast variants [20,21,22], provide a mainstream solution by extending an additional spectral dimension to disperse acquired signals. Recently, spectral-editing MRS techniques based on singlet states [23,24,25,26] also enable the extraction of targeted *J*-coupled metabolites from crowded NMR resonances. However, the singlet-state MRS method requires prior knowledge of coupled proton pairs, and only the signals from targeted coupled proton pairs are acquired in a single scan, which may obstruct their further applications. Alternatively, pure-shift NMR techniques [27,28,29,30,31], which collapse *J*-coupling splittings and preserve only chemical shift information in the singlet peak manner, offer an efficient manner to enhance spectral resolution and serve for satisfactory measurements of complex chemical and biological samples and have seen broad application scenarios in various branches of chemistry [32,33,34,35,36,37]. Considering the spectral congestion challenge faced by researchers in MRS studies, a demand for high-resolution methodologies based on pure-shift NMR has arisen in practical applications to biological samples.

**Figure 1:**
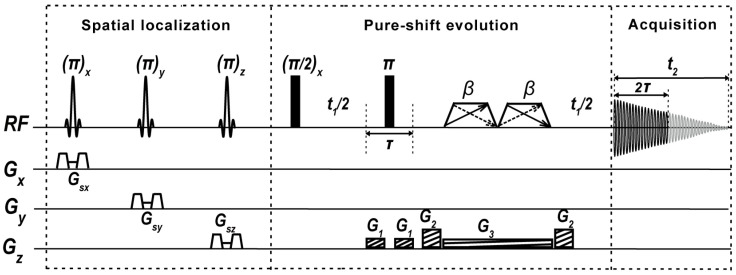
Pulse-sequence diagram of PSYCHE-ISIS MRS experiments. Sinc-shaped pulses denote π slice-selection RF pulses, along with three orthogonal slice-selective gradients, *G_sx_*, *G_sy_*, and *G_sz_*, used for the spatial volume localization. Thin and fat black bars are π/2 RF non-selective pulses. The trapezoids, including double opposed-direction arrows describing two frequency-sweep directions, indicate small-flip-angle (*β*) saltire chirp pulses; *G*_1_ and *G*_2_ are coherence selection gradients; *G*_3_ is a weak gradient matching with chirp pulses; *t*_1_ is the indirect evolution period, and *t*_2_ is the direct acquisition period; and 2*τ* the time interval of pure-shift chunks.

In this study, we present a pure-shift-based localized ^1^H MRS method, named Pure Shift Yielded by Chirp-Excitation-based Image-Selected In vivo Spectroscopy (PSYCHE-ISIS), shown in Figure 1, for high-resolution measurements of biological samples. The PSYCHE-ISIS method is designed based on the combination of the spatial localization scheme, ISIS [12], and the pure-shift NMR scheme, PSYCHE [29], to record 1D pure-shift MRS spectra from the targeted localization volume. PSYCHE-ISIS exploits the concept of pure-shift NMR to MRS measurements and is well-suited to MRS studies of biological samples in MRI devices. The feasibility and applicability of the proposed MRS method are evaluated by experiments on phantom samples and in vitro biological samples at a 7.0 T animal MRI scanner.

## 2. Results

### 2.1. Two-Compartment Phantom

**Figure 2:**
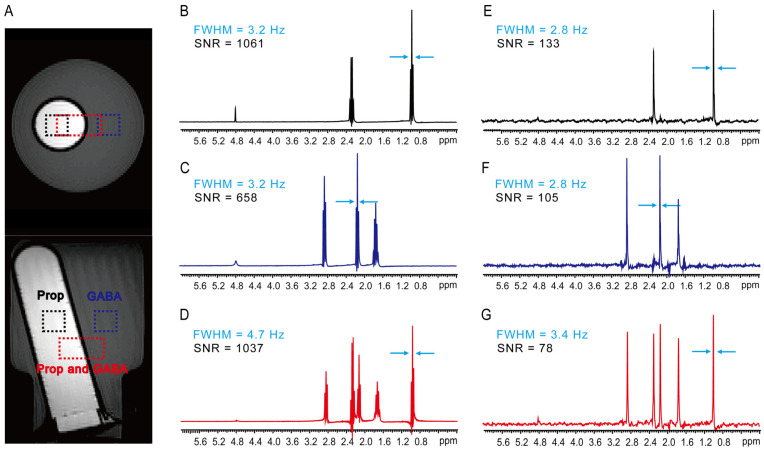
MRS experiments on a two-compartment phantom containing 1.0 M Prop (inner bottle) and 1.0 M GABA (outer bottle) aqueous solutions. (**A**) Axial and coronal spin-echo MRI images of the two-compartment phantom, black dash squares show the localized volume of 5 × 5 × 5 mm^3^ in the inner bottle, blue dash squares show the localized volume of 5 × 5 × 5 mm^3^ in the outer bottle, and red dash squares show the localized volume of 5 × 10 × 5 mm^3^ in both inner and outer bottles. (**B**–**D**) 1D PRESS spectra acquired from localized volumes in the inner bottle (**B**), the outer bottle (**C**), and both bottles (**D**). (**E**–**G**) 1D PSYCHE-ISIS spectra acquired from localized volumes in the inner bottle (**E**), the outer bottle (**F**), and both bottles (**G**). Spectral resolution is calculated according to the full widths at half maximum (FWHM) of selected peaks marked by blue arrows in all spectra.

PSYCHE-ISIS is first performed on a two-compartment phantom built with two different plastic bottles, filled with 1.0 M Propionate (Prop in the inner bottle) and γ-aminobutyric acid (GABA in the outer bottle) aqueous solution, to demonstrate its feasibility and performance on accurate spatial volume localization and desired pure-shift spectra extraction (Figure 2). In this experiment, two MRS techniques, the proposed PSYCHE-ISIS and the conventional PRESS, are adopted for comparison. Spin-echo MRI images from axial and coronal orientations visually show two-compartment phantom and spatially localized volumes for MRS experiments (Figure 2A). By reference to spin-echo MRI images with localized volumes, PRESS and PSYCHE-ISIS enable accurate acquisition of MRS spectra from different localized phantom parts. Figure 2B,E show 1D PRESS MRS spectra and 1D PSYCHE-ISIS MRS spectra from the Prop in the inner bottle (5 × 5 × 5 mm^3^ marked with the black dash square), respectively. Figure 2C,F show 1D PRESS spectra and 1D PSYCHE-ISIS spectra from GABA in the outer bottle (5 × 5 × 5 mm^3^ marked with the blue dash square). Figure 2D,G show 1D PRESS spectra and 1D PSYCHE-ISIS spectra from Prop and GABA in both inner and outer bottles (5 × 10 × 5 mm^3^ marked with the red dash rectangle). All these MRS spectra properly give the desired signals from localized volumes of interest without other undesired signal contaminations, and this confirms the performance of accurate volume localization by PSYCHE-ISIS, similar to the conventional PRESS. Different from multiplet peaks observed in the 1D PRESS spectra (Figure 2B–D), all observed peaks in the PSYCHE-ISIS spectra (Figure 2E–G) are simplified into singlets because of *J*-coupling elimination by the PSYCHE pure-shift scheme. Although *J* couplings are beneficial to structural analysis and give aid to chemical assignments, *J*-coupling modulation during the spatial localization process of PRESS experiments leads to phase distortions in the resulting 1D PRESS spectra. For example, the multiplet peaks at 0.98 ppm and 2.30 ppm suffer from phase distortions, and they are presented as phase-twist lineshapes in the 1D PRESS spectrum of mixed Prop and GABA (Figure 2D), and it is generally difficult to correct this phase distortion by standard phase correction operation. By contrast, due to the volume localization before magnetization excitation by the ISIS localization scheme, PSYCHE-ISIS directly avoids the influence of *J*-coupling modulation and delivers the satisfactory 1D MRS spectrum with decent in-phase lineshapes. Benefitting from the combined actions of the PSYCHE pure-shift and the ISIS localization schemes, PSYCHE-ISIS records the desired pure-shift MRS with a slight resolution enhancement compared to the PRESS, thus facilitating distinct chemical shift assignments. Resolution comparisons between PSYCHE-ISIS and PRESS spectra are also performed. For MRS experiments on a smaller localized volume of 5 × 5 × 5 mm^3^, the spectral resolution in the 1D PSYCHE-ISIS spectra is 2.8 Hz (Figure 2B,C), while the spectral resolution in the PRESS is 3.2 Hz. For experiments on a larger localized volume of 5 × 10 × 5 mm^3^, the spectral resolution in PSYCHE-ISIS and PRESS spectra is 3.4 Hz and 4.7 Hz, respectively. The increased localized volume size would decrease the field homogeneity, and then the spectral resolution in MRS experiments on larger localized volumes decreases accordingly. Because of intrinsic signal intensity losses by the PSYCHE pure-shift evolutions, PSYCHE-ISIS regrettably suffers from a lower signal-to-noise ratio (SNR) of about an order of magnitude compared to the PRESS, and this is a prevalent problem in pure-shift NMR applications. Despite that, the proposed PSYCHE-ISIS suggests a feasible way to enable the implementation of pure-shift MRS measurements in higher spectral resolution.

### 2.2. Brain Metabolite Phantom

**Figure 3:**
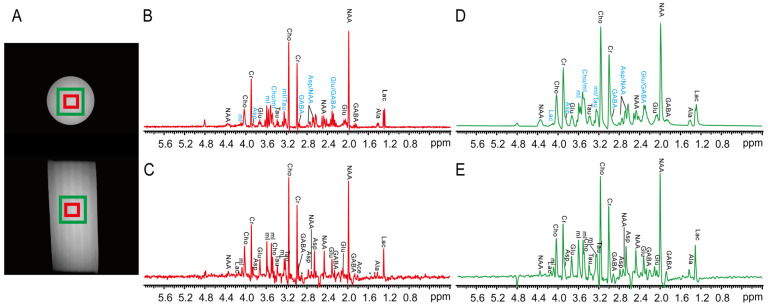
MRS experiments on brain metabolite phantom. (**A**) Axial and coronal spin-echo MRI images for this phantom sample; red rectangles indicate the smaller localized volume of 5 × 5 × 5 mm^3^, and green rectangles show the larger localized volume of 12 × 12 × 12 mm^3^. (**B**,**C**) 1D MRS spectra acquired from the smaller localized volume by PRESS and PSYCHE-ISIS, respectively. (**D**,**E**) 1D MRS spectra acquired from the larger localized volume by PRESS and PSYCHE-ISIS, respectively. Assigned metabolites are shown in all 1D MRS spectra, and some indistinctly assigned peaks in 1D PRESS spectra (**B**,**D**) are marked by blue. The asterisks (*) denote the unassigned resonances or spectral artifacts.

To further show the applicability of PSYCHE-ISIS on complex samples exhibiting crowded resonances in 1D NMR, we perform MRS experiments on a brain metabolite phantom that contains various metabolites (Figure 3). As shown in axial and coronal MRI images (Figure 3A), two localized volumes of 5 × 5 × 5 mm^3^ (marked by red square) and 12 × 12 × 12 mm^3^ (marked by green square) are selected for PRESS and PSYCHE-ISIS experiments. Figure 3B,C show 1D PRESS and 1D PSYCHE-ISIS spectra acquired from the smaller localized volumes of 6 × 6 × 6 mm^3^, respectively. Figure 3D,E show 1D PRESS and 1D PSYCHE-ISIS spectra acquired from the larger localized volumes of 12 × 12 × 12 mm^3^, respectively. Due to abundant metabolites, along with their limited chemical shift dispersion ranges and extensive *J*-coupling multiplets, spectral congestion, even peak overlapping, is observed in the resulting 1D PRESS spectra (Figure 3B,D), thus rendering peak assignments and metabolite identifications challenging. For example, myo-inositol (m-Ins) peaks and choline chloride (Cho) are crowded together at ~3.26 ppm, and it is difficult to distinguish these two metabolite peaks, and the observed multiplet peaks are jointly assigned to mI/Cho. Similarly, the overlapped multiplet peaks from myo-inositol and taurine at ~3.22 ppm are assigned to mI/Tau, and the crowded multiplet peaks from N-acetyl aspartate (NAA) and aspartate (Asp) at ~2.75 ppm are assigned to NAA/Asp. Additionally, some weak multiplet peaks from low-concentration metabolites are covered by nearby strong peaks, and they are generally unable to assign, such as mI at ~4.09 ppm, Asp at ~3.86 ppm, and GABA at ~2.97 ppm. Compared to 1D PRESS spectra from the smaller localized volume (Figure 3B), spectral resolution is degraded in the 1D PRESS spectra from the larger localized volume (Figure 3D) because of degenerative field homogeneity in the larger localized volume. This further blurs multiplet peaks, particularly crowded multiplet peaks, and imposes an obstruction on subsequent metabolite analysis. Fortunately, PSYCHE-ISIS delivers the 1D pure-shift spectra (Figure 3C,E) with a decent resolution gain by simplifying overlapped multiplets into resolved singlets; therefore, it can explicitly disentangle these observed crowded peaks and benefit unambiguous peak assignments. According to previous reports [38,39], singlet peaks observed in the 1D PSYCHE-ISIS spectra are distinctly assigned to corresponding metabolites according to their chemical shifts, and the assignment results are summarized in Table S2. Meaningfully, some low-concentration metabolites located in crowded spectral regions, e.g., Asp and GABA located at ~3.86 ppm and ~2.97 ppm, initially ignored in the PRESS spectra (Figure 3B,D), are readily recovered and identified in the pure-shift MRS spectra by PSYCHE-ISIS (Figure 3C,E). Although PSYCHE-ISIS still suffers from the lower SNR caused by the intrinsic signal intensity losses in pure-shift evolution, this lower SNR performance to some content is compensated by the collapse of multiplets into singlet peaks. Additionally, a relatively large localized volume can be set to enhance the SNR in PSYCHE-ISIS experiments while maintaining the acceptable spectral resolution, shown as the 1D PSYCHE-ISIS spectra from the larger localized volume in Figure 3E. As a consequence, the PSYCHE-ISIS protocol serves as a useful vehicle for high-resolution MRS measurements of complex metabolites that contain crowded or even overlapped NMR resonances.

### 2.3. In Vitro Pig Brain Tissue

**Figure 4:**
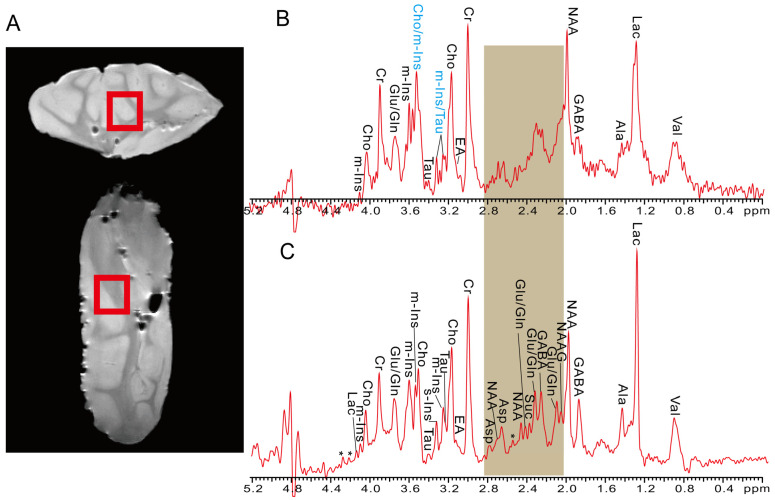
MRS measurements on intact pig brain tissues. (**A**) Axial and coronal spin-echo MRI images for this pig brain tissue sample and the spatially localized volume of 10 × 10 × 10 mm^3^ shown in the red square. (**B**) 1D PRESS MRS spectrum. (**C**) 1D PSYCHE-ISIS MRS spectrum acquired from the spatially localized volume. Assigned metabolites are shown in both 1D MRS spectra, and some indistinctly assigned peaks in 1D PRESS spectrum (**B**) are marked by blue. And some almost indistinguishable peaks in the brown spectral region of 1D PRESS MRS are recovered and assigned in 1D PSYCHE-ISIS MRS. The asterisks (*) denote the unassigned resonances or spectral artifacts.

In this section, PSYCHE-ISIS is tested on in vitro pig brain tissues to show its applicability to biological sample measurements with the challenge of lower metabolite concentrations and field inhomogeneity caused by magnetic susceptibility variations inside tissues (Figure 4). In the MRS experiments, the intact pig brain tissue is directly packed and put into the scanner without further sample pretreatment. Spin-echo MRI images indicating the packed pig brain tissue sample and targeted localized volumes for MRS experiments are shown in Figure 4A. For conventional PRESS measurements (Figure 4B), the resulting 1D MRS spectrum faces the challenge of spectral congestion caused by abundant metabolites with extensive *J*-coupling multiplet structures and residual field inhomogeneity originating from intrinsic magnetic susceptibility variations, thus impeding identification and extraction of metabolites, such as Cho/m-Ins and m-Ins/Tau marked by the blue in Figure 4B. Additionally, due to the degraded spectral resolution, some observed peaks in the brown spectral regions are almost indistinguishable, and it is difficult to perform assignments for these peaks in this 1D PRESS spectrum (Figure 4B). By contrast, the proposed PSYCHE-ISIS presents an effective alternative to record 1D pure-shift MRS spectra with enhanced spectral resolution, thus elevating biological detections and metabolite analyses. Benefitting from the spectral simplification from multiplet to singlet peaks, all observed peaks are relatively highlighted from spectral congestion in the 1D PSYCHE-ISIS spectrum (Figure 4C). Although inhomogeneous line broadenings caused by intrinsic field inhomogeneity inside biological tissues still exist, it can readily perform metabolite assignments for these in vitro pig brain tissues. For example, some multiplet peaks from Cho, Tau, and mI, which are ambiguous in the 1D PRESS spectrum, can clearly distinguished and identified. More meaningfully, low-concentration metabolites with multiplet structures are highlighted, resulting from the pure-shift simplification with intensive singlet peaks. Because of the decent resolution gain, useful spectral information in the brown regions is recovered, and related metabolites can be assigned. Referring to previous literature [30,40], well-resolved peaks in the 1D PSYCHE-ISIS spectra are assigned to specific functional groups of metabolites, and assigned groups and corresponding chemical shifts are summarized in Table S3. In addition, PSYCHE-ISIS MRS measurements of the other in vitro biological samples of intact grape tissues that contain extremely crowded and overlapped NMR resonances are also performed; the experimental results are given in Figure S2 of Supplementary Materials. Therefore, the performance of PSYCHE-ISIS for high-resolution probing of in vitro biological samples is self-evident.

## 3. Discussion

As demonstrated with the aforementioned results, compared with the classical localized 1D ^1^H MRS methods, such as PRESS, the pure-shift-based localized ^1^H MRS methods, here PSYCHE-ISIS as a proof of concept, enable the accurate metabolite measurements on the selected volumes of interest positioned by reference to the anatomical structure in MRI. The detailed performance comparison between the proposed PSYCHE-ISIS method and the PRESS method is summarized in Table 1. As summarized in Table 1, compared to the conventional PRESS MRS method, the proposed PSYCHE-ISIS provides spectral resolution enhancement indicated by the full widths at half maximum (FWHM) of selected spectral peaks, but it generally suffers from the lower signal-noise-ratio (SNR) of about an order of magnitude. Nevertheless, the lower SNR performance to some content is compensated by the collapse of multiplets into singlet peaks, especially for low-concentration metabolites with complex multiplet structures. In particular, benefitting from the removal of *J* couplings, PSYCHE-ISIS allows one to recover and resolve key metabolite molecules initially submerged in the spectral congestions in conventional MRS and delivers high-resolution measurements of in vitro biological tissues containing extensive metabolites and overlapped NMR resonances, thus elevating metabolite analysis and potential disease diagnosis. Additionally, in contrast to conventional PRESS and STEAM methods suffering from *T*_2_-relaxation weighting and *J*-coupling modulation, PSYCHE-ISIS implements the spatial localization before magnetization excitation and hardly introduces the influences on subsequent pure-shift extraction, thus delivering decent in-phase peaks and more applicable to long TE or short *T*_2_-relaxation cases.

Noting that since the *J* couplings also serve as important tools for molecular identification, simple extensions of localized pure-shift 2D*J* [37,39] by incorporating the echo-train *J*-acquisition module during the acquisition periods would allow one to directly refurnish the *J*-coupling information along the orthogonal dimension, and deliver resulting in-phase localized pure-shift 2D*J* spectra without an additional significant increase in experiment times. Similarly, according to practical application requirements and scenarios, the proposed localized pure-shift ^1^H MRS protocols can be readily extended to combine the advantages of pure-shift and localized 2D MRS [41] and implement desired pure-shift-based localized 2D MRS, such as pure-shift-based LCOSY [22], presenting more metabolite structure and kinetic information contents.

Despite with the aforementioned gains, the limitations of pure-shift-based localized MRS still need to be discussed. First, signal losses during the PSYCHE pure-shift evolution would constitute the primary limitation factor for broader detections, thus generally requesting increasing scan numbers to accumulate signals, and thus, improvement in PSYCHE-ISIS with higher SNR is still demanded for practical applications in disease diagnosis or in vivo measurements. Nevertheless, among the pure-shift methodology members, the PSYCHE module preserves the highest signal intensities, and the use of optimized “saltire chirp” pulses further gives a four-fold sensitivity enhancement with the same spectral purity [31]. Also, advanced iterative algorithms [42,43] and popular deep learning proposals [44,45,46] have proved the powerfulness for NMR denoising, thus presenting the possibility of endowing satisfactory MRS with fewer accumulation scans. Additionally, the sensitivity performance would also benefit a lot from advanced instruments equipped with cryoprobes and higher magnetic fields [47,48]. Second, the pseudo-2D signal acquisition for pure-shift evolutions indicates increasing experimental times in the PSYCHE-ISIS measurements. Thus, an optimized non-uniform sampling schedule and related spectral reconstruction would contribute to alleviating this issue [49,50,51,52]. Also, the real-time ZS [28] provides another pure-shift-based localized ^1^H MRS proposal with instant homonuclear broadband decoupling, even though suffering from possible decoupling artifacts and more severe signal loss. Moreover, the accelerated pure-shift implementation may also provide compatibility against dynamic nuclear polarization techniques [53], which offer prominent sensitivity enhancement [54,55,56]. Moreover, due to the employment of ISIS spatial localization, the number of transient scans must be an integer multiple of eight to provide decent volume selection and cancel signal contamination. Fortunately, the necessary transition scans are exactly required for phase cycling of pure-shift evolution and signal accumulation to compensate for sensitivity loss.

## 4. Materials and Methods

The pulse sequence for PSYCHE-ISIS experiments is shown in Figure 1. This sequence directly accommodates the ISIS spatial single-voxel localization scheme into the PSYCHE pure-shift evolution scheme. These two schemes are implemented without mutual influence on signal evolution and achieve a satisfactory fusion for recording 1D pure-shift MRS spectra. First, the ISIS spatial localization module, composed of three slice-selective refocusing π RF pulses in union with three-dimensional orthogonal slice-selective gradients *G_sx_*, *G_sy_*, and *G_sz_* along with their corresponding crusher gradient pairs, implements accurate cube volume localization prior to magnetization excitation. Compared to conventional single-voxel localization schemes of PRESS and STEAM that perform spatial volume localization after signal excitation, ISIS achieves volume localization performance prior to signal excitation, and it would not introduce the influences on the following pure-shift evolution by the PSYCHE scheme. Therefore, ISIS is more suitable to long *TE* cases, e.g., pure-shift evolution with a relatively long evolution period, and it also yields more decent in-phase peaks. After the spatial single-voxel localization by ISIS, the sequence starts with a non-selective π/2 RF pulse for signal excitation. Subsequently, the PSYCHE pure-shift element, composed of a non-selective π refocusing pulse and a couple of small-angle (*β*) “saltire” frequency-sweep (chirp) pulses [31] along with the weak *z*-axial gradient G*_3_* centered in symmetric pure-shift evolution periods *t*_1_/2, and two pairs of coherence selection gradients *G*_1_ and *G*_2_, aims at the implementation of pure-shift evolution and extraction. Among broadband pure-shift decoupling families, the PSYCHE module, which preserves the highest signal intensities and superior spectral purity, as well as tolerance of strong couplings existing in certain metabolite molecules, is adopted to endow the satisfactory spectral resolution for the extraction of the metabolite information from overlapping resonances. After the pseudo-2D signal acquisition and subsequent pure-shift chunk concentration common to pure-shift NMR techniques, the desired high-resolution pure-shift ^1^H MRS is obtained. Due to the localization before signal excitation, the two functional schemes of spatial localization and pure shift are independent of each other, indicating the generality of different pure-shift and spatial localization modules and the convenience for experimental setup and pulse-sequence transplantation. Detailed theoretical derivation for signal evolution of PSYCHE-ISIS, including the ISIS spatial localization mechanism (Table S1 and Figure S1) and the PSYCHE pure-shift evolution, is given in the Supporting Information.

To demonstrate the versatility and applicability of the proposed PSYCHE-ISIS on recording pure-shift MRS spectra from targeted localized volumes, we chose two types of samples for experimental proofs: (a) phantom samples, including a two-compartment phantom with two separated metabolites and a brain metabolite phantom with crowded resonances, and (b) in vitro biological samples, namely intact pig brain and grape tissues, that contain abundant metabolite compositions and suffer from spectral congestion. Biological samples (intact pig brain tissues and grape tissues) used in our experiments were approved by the Institutional Review Board at Xiamen University, Xiamen, China (XMULAC20230182). All experiments were carried out on a Varian 7.0 T small animal MRI scanner (Palo Alto, CA, USA) equipped with a 160 mm inner bore diameter and a 63/95 mm quad birdcage coil. The system was equipped with a gradient coil system producing a maximum gradient strength of 40 G/cm, and the detection probe was well-tuned to preserve high sensitivity. Detailed experimental settings are given in Supplementary Materials.

## 5. Conclusions

In this work, a pure-shift-based localized ^1^H MRS approach, namely PSYCHE-ISIS, is exploited and demonstrated to implement the resolution-enhanced MRS measurements on the targeted volumes via incorporating the PSYCHE pure-shift elements into the ISIS spatial localization. Benefiting from the simplification from multiplets to singlets, the pure-shift-based localized ^1^H MRS alleviates the spectral congestions in conventional MRS and enables the identification of metabolites initially submerged in the overlapped resonances. Experimental results verify that the localized pure-shift ^1^H MRS protocol delivers high-resolution measurements of biological samples containing extensive metabolites and exhibiting overlapped NMR resonances, although somewhat limited to degraded signal intensity and acquisition efficiency. As a consequence, this study provides an effective MRS methodology for metabolite analysis and potential in vivo detections.

## Figures and Tables

**Table 1:** Detailed performance comparison between the proposed PSYCHE-ISIS and PRESS.

	Performance Terms	Spectral Resolution	Signal-to-Noise Ratio (SNR)	Metabolite Detection Capabilities
Samples				
The two-compartment phantom	Prop (Figure 2B,C)	3.2 Hz/2.8 Hz ^a^	1061/133	Yes/Yes
	GABA (Figure 2D,E)	3.2 Hz/2.8 Hz	658/105	Yes/Yes
	Prop and GABA (Figure 2F,G)	4.7 Hz/3.4 Hz	1037/78	Yes/Yes
Brain metabolite phantom	5 × 5 × 5 mm^3^ (Figure 3B,C)	3.5 Hz/3.3 Hz ^b^	175/38	No/Yes ^d^
	12 × 12 × 12 mm^3^ (Figure 3D,E)	8.6 Hz/5.3 Hz	984/140	No/Yes ^d^
Pig brain tissues (Figure 4B,C)		9.8 Hz/7.8 Hz ^c^	238/71	No/Yes ^d^

^a^ 3.2 Hz/2.8 Hz denote the full widths at half maximum (FWHM) of selected peaks acquired by the conventional PRESS and proposed PSYCHE-ISIS. ^b^ The FWHM of PSYCHE-ISIS for the small volume (5 × 5 × 5 mm^3^) is calculated by the Cho peak located at ~3.16 ppm, and the others are calculated by the NAA peak located at ~1.98 ppm. ^c^ The FWHMs are calculated by the Cr peak located at ~3.00 ppm. ^d^ No denotes that the conventional PRESS is not applicable to detecting metabolites located in crowded regions, such as peaks marked in blue in Figure 3 and Figure 4.

## Data Availability

All data needed to evaluate the conclusions in this paper are present in the paper and/or the Supplementary Materials. Additional data related to this paper may be requested from the corresponding authors.

## Supplementary Materials

The supporting information can be downloaded at https://www.mdpi.com/article/10.3390/ijms25094698/s1. References [12,57,58] are cited in the supplementary materials.
