# Supplementary material for: Pure-Shift-Based Proton Magnetic Resonance Spectroscopy for High-Resolution Studies of Biological Samples
Source: Int J Mol Sci. 2024 Apr 25;25(9):4698. doi: 10.3390/ijms25094698 (PMC11083948; doi:10.3390/ijms25094698)
Supplement: Supplementary file 1 [file ijms-25-04698-s001.zip › ijms-2966910-supplementary.pdf]

Supplementary Materials

Pure-Shift-Based Proton Magnetic Resonance Spectroscopy for High-Resolution Studies of Biological Samples

Haolin Zhan[1,2], Yulei Chen[1], Yinping Cui[1], Yunsong Zeng[1], Xiaozhen Feng[1], Chunhua Tan[1], Chengda Huang[1], Enping Lin[1], Yuqing Huang[1,*], and Zhong Chen[1]

[1] Department of Electronic Science, Fujian Provincial Key Laboratory of Plasma and Magnetic Resonance, State Key Laboratory of Physical Chemistry of Solid Surfaces, Xiamen University, Xiamen, 361005, China.

[2] Department of Biomedical Engineering, Anhui Provincial Engineering Research Center of Semiconductor Inspection Technology and Instrument, Anhui Province Key Laboratory of Measuring Theory and Precision Instrument, Hefei University of Technology, Hefei, 230009, China.

* Correspondence: yqhuangw@xmu.edu.cn (Y. Q. Huang).

1、Methodology

Table S1 Experimental setups of ISIS localization[1] prior to magnetization excitation

Transient scan number	*x*-selective pulse	*y*-selective pulse	*z*-selective pulse	Contribution to total spectrum (C_i)[b]
1	OFF[a]	OFF	OFF	**+1**
2	ON[a]	OFF	OFF	-1
3	OFF	ON	OFF	-1
4	ON	ON	OFF	**+1**
5	OFF	OFF	ON	-1
6	ON	OFF	ON	**+1**
7	OFF	ON	ON	**+1**
8	ON	ON	ON	-1

[a] OFF/ON denotes the use/absence of the selective pulses for certain experimental scan.

[b] C_i denotes the contribution coefficient of the *i*-th scans

Initially, the ISIS localization scheme combines NMR signals from 8 transient scans whose experimental setups are summarized in Table 1, to enable accurate selection of targeted volume cube and achieve adaptive suppression on all signals except those in the select cube. Considering a general case, desired signals S_R marked in red squares indicated in Figure S1 and unwanted signals S_B and S_G in blue and green squares from the coaddition of the eight transient scans are described in the following Equations (1-3).

$$S_B = C_1 + C_2 \bullet \cos x + C_3 + C_4 \bullet \cos y + C_5 \bullet \cos z + C_6 \bullet \cos x \cos z + C_7 \bullet \cos z + C_8 \bullet \cos x \cos z, \quad \text{(S1)}$$

$$S_G = C_1 + C_2 + C_3 \bullet \cos y + C_4 \bullet \cos y + C_5 \bullet \cos z + C_6 \bullet \cos z + C_7 \bullet \cos y \cos z + C_8 \bullet \cos y \cos z, \quad \text{(S2)}$$

$$S_R = C_1 + C_2 \bullet \cos x + C_3 \bullet \cos y + C_4 \bullet \cos x \cos y + C_5 \bullet \cos z + C_6 \bullet \cos x \cos z + C_7 \bullet \cos y \cos z + C_8 \bullet \cos x \cos y \cos z, \quad \text{(S3)}$$

in which *x*, *y* and *z* signify the spin nutation angles of slice-selective pulses along three axes, respectively. To ensure the elimination of signals coming from surrounding

volume regions around the cubes of interest, namely S_B and S_G for all spin nutation angles, the contribution coefficients need to be simultaneously satisfied as follows:

$$C_1 + C_3 = C_2 + C_4 = C_5 + C_7 = C_6 + C_8, \tag{S4}$$

and

$$C_1 + C_2 = C_3 + C_4 = C_5 + C_6 = C_7 + C_8. \tag{S5}$$

As a consequence, the contribution coefficients are adopted as those depicted in the last column of Table 1 to suppress on all signals except those in the selected cube (marked by the red), and the overall signal intensity *S* from the volume cubes of interest is retained as follows:

$$S = 1 - \cos x - \cos y + \cos x \cos y - \cos z + \cos x \cos z + \cos y \cos z - \cos x \cos y \cos z. \tag{S6}$$

For a common case that three slice-selective refocusing π pulses along three orthogonal axes are adopted as indicated in Figure 1, namely $x=y=z=\pi$, eight times overall signals contributed from the volume cube of interest are acquired compared to those in a single-scan acquisition, namely $S=8$, which is equivalent to a signal average of eight performed on the central cube in isolation. More meaningfully, the cancellation on unwanted signals from surrounding 3D space occurs independent of the spin nutation angles of used slice-selective pulses, thus offering robust 3D volume localization against RF field (B1) inhomogeneity (in other words, imperfect pulse angles) and guaranteeing high-purify targeted signals.

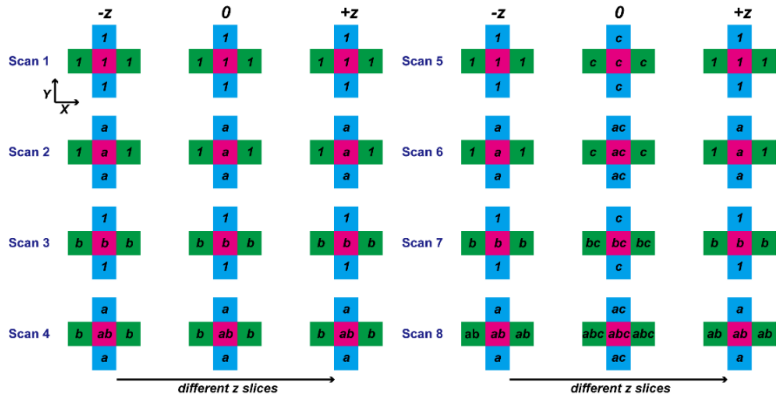

Figure S1. The 3D spatial localization diagram for the ISIS module. The targeted 3D cube is mark by the red boxes, while the blue and green boxes refers to the surrounding volume regions around the cubes of interest. ***a***, ***b***, ***c*** denote cos *x*, cos *y*, and cos *z* modulated from the *x*, *y*, and *z*-selective pulses with the spin nutation angles of *x*, *y*, and *z*, respectively.

After the spatial localization prior to excitation, a non-selective π/2 RF pulse flips the magnetization within the selected volume cube from the *z* direction into *xy* plane. To intuitively understand the signal evolution, the raising and lowering operators are used in the following description. For simplification, an AX spin-1/2 weakly-coupled system is considered, in which *A* and *X* denote spins *I* and *S* coupled by the coupling constant J_{IS}, and only the evolution of spin *I* is traced, since spins *I* and *S* shares the similar evolutions. Because only I^- signals can be observed during the acquisition, the effective coherence path is traced as:

$$I_z \xrightarrow{90^\circ I_x} I^- \xrightarrow{180^\circ I_x} I^+ \xrightarrow{\beta\ chirp} I_z \xrightarrow{\beta\ chirp} I^- . \tag{S7}$$

Thus, experiencing the first $t_1/2$ pure shift evolution periods, signals evolve as follows:

$$\sigma_0 = I_z \xrightarrow{\frac{\pi}{2} I_x} \frac{1}{2i} I^- \xrightarrow{\Omega_I I_z \frac{t_1}{2} + \pi J_{IS} \frac{t_1}{2} \cdot 2I_z S_z} \sigma_1 = \frac{1}{2i} I^- e^{i\Omega_I \frac{t_1}{2}} \left[\cos(\pi J_{IS} \frac{t_1}{2}) + i2S_z \sin(\pi J_{IS} \frac{t_1}{2}) \right] \tag{S8}$$

Before a couple of small-angle (β) chirp pulses are applied, the signals evolve as

follows:

$$
\begin{aligned}
I_z &\xrightarrow{\frac{\pi}{2}I_x} \frac{1}{2i}I^- \xrightarrow{\Omega_I I_z \frac{t_1}{2}+\pi J_{IS}\frac{t_1}{2}\cdot 2I_z S_z} \frac{1}{2i}I^- e^{i\Omega_I \frac{t_1}{2}}\left[\cos(\pi J_{IS}\frac{t_1}{2})+i2S_z\sin(\pi J_{IS}\frac{t_1}{2})\right] \\
&\xrightarrow{\tau=T/2=1/4\mathrm{SW1}} \frac{1}{2i}e^{i\Omega_I\tau}I^- e^{i\Omega_I \frac{t_1}{2}}\left[\cos(\pi J_{IS}\frac{t_1}{2})+i2S_z\sin(\pi J_{IS}\frac{t_1}{2})\right][\cos(\pi J_{IS}\tau)+i2S_z\sin(\pi J_{IS}\tau)] \\
&\xrightarrow{\pi(I_x+S_x)} \frac{1}{2i}e^{i\Omega_I\tau}I^+ e^{i\Omega_I \frac{t_1}{2}}\left[\cos(\pi J_{IS}\frac{t_1}{2})-i2S_z\sin(\pi J_{IS}\frac{t_1}{2})\right][\cos(\pi J_{IS}\tau)-i2S_z\sin(\pi J_{IS}\tau)] \\
&\xrightarrow{\tau=1/4\mathrm{SW1}} \sigma_2 = \frac{1}{2i}I^+ e^{i\Omega_I \frac{t_1}{2}}\left[\cos(\pi J_{IS}\frac{t_1}{2})-i2S_z\sin(\pi J_{IS}\frac{t_1}{2})\right][\cos(\pi J_{IS}2\tau)\text{-}i2S_z\sin(\pi J_{IS}2\tau)] \\
&= \frac{1}{2i}I^+ e^{i\Omega_I \frac{t_1}{2}}\{\cos[\pi J_{IS}(\frac{t_1}{2}+2\tau)]-2iS_z\sin[\pi J_{IS}(\frac{t_1}{2}+2\tau)]\}.
\end{aligned}
\tag{S9}
$$

Then, as a couple of chirp pulses flip the spin state of the observed nucleus *I* while preserving the spin state of the coupled nucleus *S* unchanged, Under a small-angle approximation, the observed signals evolve into:

$$\sigma_3 = A\beta^2 I^- e^{i\Omega_I \frac{t_1}{2}}\{\cos[\pi J_{IS}(\frac{t_1}{2}+2\tau)]-2iS_z\sin[\pi J_{IS}(\frac{t_1}{2}+2\tau)]\}, \tag{S10}$$

in which *A* denotes the coefficients not directly related to effective signal expressions. After the second $t_1/2$ evolutions, the effective signals are given as:

$$\sigma_4 = A\beta^2 I^- e^{i\Omega_I t_1}[\cos(\pi J_{IS}2\tau)+2iS_z\sin(\pi J_{IS}2\tau)], \tag{S11}$$

in which the *J* coupling evolutions and modulations during t_1 are suppressed, and only *J* coupling evolutions during a short constant time T=2τ still exist. During the following signal acquisition t_2, the observable signals are traced as

$$\sigma_5 = A\beta^2 I^- e^{i\Omega_I t_1}e^{i\Omega_I t_2}[\cos(\pi J_{IS}(t_2-2\tau))],\ \ t_2\in[0,4\tau]. \tag{S12}$$

Then, a series of data chunks of 1/SW1 (2T=4τ) duration with incremental variables t_1 are sequentially concatenated to reconstruct desired 1D pure shift, and *J* couplings are completely refocused in the middle of each data chunk. With a reasonable approximation that when the time length of each data chunk is less than 20 ms, the *cos* term is close to 1, after the pure shift reconstruction, the resulting signals from *I* spin

are given as:

$$\sigma_6 = A\beta^2 I^- e^{i\Omega_i t}, \tag{S13}$$

in which $t=t_1+t_2$. As a consequence, benefiting from the pure shift element and two symmetric $t_1/2$ evolutions, the chemical shift evolution is preserved while the *J* coupling evolution is refocusing, thus removing *J* coupling splittings, suppressed coupling modulations as well as implementing desired pure shift.

Regarding the resulting sensitivity performance, the signal intensities of PSYCHE-ISIS method mainly depend on the used PSYCHE pure shift element, thus preserving the most magnetization among pure shift experiments. As shown in Eq. (13), the preserved signal intensities of resulting spectra are approximatively proportional to β^2, where β is the flip angle of chirp pulses. For common chirp pulses used in PSYCHE element, the ratio between the desired pure shift signals and unwanted artifact signals, namely spectral purity is dependent on $1/\beta^2$. Herein, optimized "saltire" chirp pulses containing two small-angle chirp pulses with opposite sweeping directions, are adopted to replace the original chirp pulses, thus implementing an improved spectral purity of $1/(\beta/2)^2$, namely $4/\beta^2$. As a result, the necessary compromise between resulting signal sensitivity and spectral purity, and in general a relatively small flip angle of β is used to ensure satisfactory pure shift measurements.

2. Experimental details

All experiments were executed on a Varian (Palo Alto, CA, USA) 7 T small animal magnetic resonance scanner at 293 K with a 130 mm inner bore diameter and a 63/95 mm quad birdcage coil, equipped with a gradient coil system and its maximum gradient strength of 40 G cm^{-1}. The variable power and optimized relaxation delays (VAPOR) module provided by the scanner was used to suppress the strong water signal. For comparison, the PRESS sequence was utilized as a reference scheme in our experiments on aqueous solution, pig brain tissue and rat *in vivo*. The methods and experiments on rat were approved by the Institutional Review Board at Xiamen University, Xiamen, China (XMULAC20230182).

A two-compartment phantom built with the plastic bottle filled with 1.0 M Propionate (Prop in inner tube) and 1.0 M γ-aminobutyric acid (GABA in outer bottle) aqueous solution was used to demonstrate the localization performance of the proposed PSYCHE-ISIS sequence. Before MRS experiments, spin-echo images on axial and coronal orientations of the plastic bottle were acquired to show the localized regions with different color panes. Three voxels were selected based on the images. A volume of 5 × 5 × 5 mm^3 was positioned in the inner plastic bottle, the other volume of 5 × 5 × 5 mm^3 was positioned in the outer plastic bottle, whereas a large volume of 5 × 10 × 5 mm^3 with green rectangle line was positioned between the inner plastic centrifuge tube and outer plastic bottle. The conventional PRESS and the pure shift PSYCHE-ISIS sequences were both applied to the three localized volume as described above for comparison purpose. For the PRESS pulse sequence, the total echo time (TE)

= 13.36 ms, the pulse repetition time TR = 2000 ms, the acquisition time = 500 ms, the spectral width SW = 3 kHz and the average number nt = 8, resulting in a total experimental time was 20 sec. For the PSYCHE-ISIS experiments, the width of the $\frac{\pi}{2}$ RF hard pulse was 73.5 μs, the number of averages was 8, the parameters for the coherence selective gradients were G_1 = 24.0 G/cm, G_2 = 38.0 G/cm and duration δ = 1.5 ms. Besides, the angle of two symmetric frequency-swept chirp pulses was 15°, the weak pulsed field gradient G_3 = 0.8 G/cm with duration 30 ms. Other parameters in the PSYCHE-ISIS experiments were as follows: The pulse repetition time was 3000 ms, the acquisition time was 500 ms, and 25 × 2500 points were acquired with spectral widths of 100 Hz × 5000 Hz (F1 × F2) in about 10.0 min.

To further show the applicability of the PSYCHE-ISIS on complex samples exhibiting crowded resonances in 1D NMR, we perform MRS experiments on a brain metabolite phantom that contains various metabolites The brain metabolite phantom was consisted of the major brain metabolites, including 50 mM N-acetyl aspartate (NAA), 45 mM creatine (Cr), 40 mM choline chloride (Cho), 40 mM DL-lactic acid (Lac), 30 mM Alanine (Ala), 35 mM Acetate (Ace), 40 mM Aspartate (Asp), 40 mM Glutamate (Glu), 35 mM γ-aminobutyric acid (GABA), 35 mM Taurine (Tau), and 45 mM myo-Inositol (mI). Prior to spectral measurement, spin-echo images on axial and coronal orientations were acquired for voxel selection, including a volume of 12 × 12 × 12 mm^3 (large volume) and a volume of 6 × 6 × 6 mm^3 (small volume). For the PSYCHE-ISIS experiments, parameters were set to the width of the hard $\frac{\pi}{2}$ RF pulse was 90.5 μs,

the number of averages was 32, the CSGs with strength G_1 = 24.0 G/cm, G_2= 38.0 G/cm and duration was 1.5 ms. The angle of chirp pulses was 18°, the weak pulsed field gradient G_3 = 0.8 G cm^{-1} with duration 30 ms, the pulse repetition time was 3000 ms, the acquisition time was 500 ms, and 24 × 2500 points were acquired with spectral widths of 100 Hz × 5000 Hz (F1 × F2) in 38.4 min. The PRESS experiment was performed in both large and small voxel as described above for comparison, and the parameters were set as TE = 14 ms, TR = 3000 ms, the acquisition time = 600 ms, the spectral width SW = 5000 Hz and the average number nt = 32 with an experimental time about 2 min.

To show the applicability of the PSYCHE-ISIS to biological samples, we perform experiments on *in vitro* pig brain tissues and grape tissues. For experiments on pig brain tissues, the intact pig brain tissue is directly packed and put into the scanner without further sample pretreatment. Spin-echo images on axial and coronal orientations were acquired for the localized volume of 10 × 10 × 10 mm^3. Parameters for PSYCHE-ISIS experiments were set as following: the width of the hard $\frac{\pi}{2}$ RF pulse was 98.5 μs, the number of averages was 64, the CSGs with strength G_1 = 24.0 G/cm, G_2= 38.0 G/cm and duration was 1.5 ms. The angle of chirp pulses was 18°, the weak pulsed field gradient G_3 = 0.8 G cm^{-1} with duration 30 ms, the pulse repetition time was 2000 ms, the acquisition time was 200 ms, and 15 × 1000 points were acquired with spectral widths of 100 Hz × 5000 Hz (F1 × F2) in 32 min. The PRESS experiment was performed in the same localized volme as described above for comparison, and the parameters were set as TE = 10 ms, TR = 2000 ms, the acquisition time = 200 ms, the spectral width SW = 5000 Hz and the average number nt = 64 with an experimental time about 2.1 min. For

MRS experiments on grape tissues, spin-echo images on axial and coronal orientations were acquired for two localized volumes, a large volume of 10 × 10 × 10 mm^3 and a small volume of 5 × 5 × 5 mm^3. PSYCHE pure shift parameters in PSYCHE-ISIS experiments on intact grape tissues are the same as those on pig brain tissues. The number of averages was 16, the pulse repetition time was 2000 ms, the acquisition time was 250 ms, and 20 × 1250 points were acquired with spectral widths of 100 Hz × 5000 Hz (F1 × F2) in 10.7 min.

3. Experimental results

Figure S2 demonstrates further applications of the PSYCHE-ISIS on high-resolution probing on biological samples, namely intact grape tissues that contain extremely crowded and overlapped NMR resonances. For the localized small volume of 5 × 5 × 5 mm^3 (red square) in anatomical MRI (Figure S2A), Figure S2B illustrates that extensive metabolites with complex molecular structure in grape samples result in congested NMR resonances in resulting PRESS spectrum. For the large volume of 10 × 10 × 10 mm^3 (green square), intrinsic magnetic susceptibility variations further broadening spectral peaks, thus impeding the extraction of desired component information (Figure S2D). By contrast, the high-resolution 1D pure shift MRS (Figure S2C and S2E) is furnished with enhanced spectral resolution, elevating metabolite assignments particularly for the overlapped region between 3.0 and 4.2 ppm. Accordingly[2, 3], all resolved peaks are assigned.

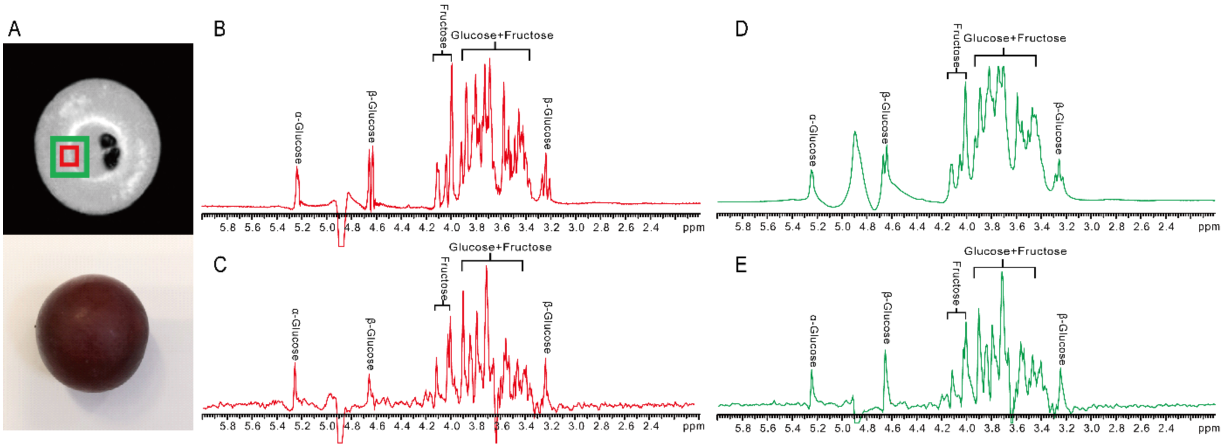

Figure S2. Experimental results on intact grape sarcocarp. (A) The anatomical MR image and a photo of the grape sample. (B, D) Localized PRESS spectra for the selected voxels of 5 × 5 × 5 mm^3 (red square) and 12 × 12 × 12 mm^3 (green square), respectively. (C, E) Localized pure shift MRS for the voxels of 5 × 5 × 5 mm^3 and 12 × 12 × 12 mm$^{3.}$

Table S2. Assigned metabolites of brain metabolite phantom obtained by the PSYCHE-ISIS

Metabolites	Groups	[1]H Chemical shifts (ppm)
Lactate (Lac)	$-CH_3$	~1.30
	-CH-	~4.13
Alanine (Ala)	$-CH_3$	~1.42
Acetate (Ace)	$-CH_3$	~1.81
γ-Aminobutyric acid (GABA)	$-CH_2-C\mathbf{H}_2-$	1.87
	$-C\mathbf{H}_2-CH_2-$	2.27
	$OOC-CH_2-$	2.97
N-Acetyl aspartate (NAA)	$-CH_3$	1.99
	$-CH-C\mathbf{H}_2-$	2.47/2.72
	$-C\mathbf{H}-CH_2-$	4.35
Glutamate (Glu)	$-CH-C\mathbf{H}_2-$	2.09
	$C\mathbf{H}_2-COO-$	2.32
	$-C\mathbf{H}-CH_2$	3.72
Aspartate (Asp)	$-CH-C\mathbf{H}_2-$	2.65/2.77
	$-C\mathbf{H}-CH_2-$	3.86
Creatine (Cr)	$N-CH_3$	3.00
	$-C\mathbf{H}_2-COO-$	3.81
Choline	$N-(CH_3)_3$	3.16
	$C\mathbf{H}_2-N-(CH_3)_3$	3.48
	$-C\mathbf{H}_2-OH$	4.03
Taurine (Tau)	$N-C\mathbf{H}_2$	3.23
	$-C\mathbf{H}_2-SO_3-$	3.39
Myo-inositol (m-Ins)	-CH-	3.25
	-CH-	3.50
	-CH-	3.59
	-CH-	4.08

Table S3. Assigned metabolites of *in vitro* pig brain tissues obtained by the PSYCHE-ISIS

Metabolites	Groups	[1]H Chemical shifts (ppm)
Valine (Val)	$-CH_3$	0.90
Lactate (Lac)	$-CH_3$	~1.28
	-CH-	~4.14
Alanine (Ala)	$-CH_3$	~1.44
γ-Aminobutyric acid (GABA)	$-CH_2$-**C**$\mathbf{H}_2$-	1.88
	-**C**$\mathbf{H}_2$-CH_2-	2.26
N-Acetyl aspartate (NAA)	$-CH_3$	1.98
	-CH-**C**$\mathbf{H}_2$-	2.46/2.70
N-Acetyl aspartate glutamate (NAAG)	$-CH_3$	2.05
Glutamate/Glutamine (Glu/Gln)	-CH-**C**$\mathbf{H}_2$-	2.09
	**C**$\mathbf{H}_2$-COO-	2.32
	-**C**$\mathbf{H}_2$-CO-NH_2	2.42
	-**CH**-CH_2	3.72
Succinate (Suc)	$-CH_2-$	2.38
Aspartate (Asp)	-CH-**C**$\mathbf{H}_2$-	2.66/2.78
Creatine (Cr)	N-CH_3	3.00
	-**C**$\mathbf{H}_2$-COO-	3.91
Ethanolamine (EA)	$-CH_2-$	3.12
Choline	N-$(CH_3)_3$	3.16
	**CH2**-N-$(CH_3)_3$	3.51
	-**CH2**-OH	4.04
Taurine (Tau)	N-**CH2**	3.23
	-**C**$\mathbf{H}_2$-SO_3-	3.40
Myo-inositol (mI)	-CH-	3.25
	-CH-	3.54
	-CH-	3.60
	-CH-	4.10
Scyllo-inositol (s-Ins)	-CH-	3.32

Reference

1. Ordidge, R.J.; Connelly, A.; Lohman, J.A.B. Image-selected in vivo spectroscopy (ISIS). A new technique for spatially selective NMR spectroscopy. *J. Magn. Reson.* **1986**, *66*, 283–294. https://doi.org/10.1016/0022-2364(86)90031-4.

2. Gilberto, M.; Maria Grazia, G.; Luca, P.; Gianni, N.; Luca, M.; Roberto, T.; Roberto, A. NMR analysis of seven selections of vermentino grape berry: metabolites composition and development. *J. Agric. Food Chem.* **2011**, *59*, 793–802.

3. Gallo, V.; Mastrorilli, P.; Cafagna, I.; Nitti, G.I.; Latronico, M.; Longobardi, F.; Minoja, A.P.; Napoli, C.; Romito, V.A.; Schäfer, H. Effects of agronomical practices on chemical composition of table grapes evaluated by NMR spectroscopy. *J. Food Compos. Anal.* **2014**, *35*, 44-52
